# First survey of Interstitial molluscs from Cayo Nuevo, Campeche Bank, Gulf of Mexico

**DOI:** 10.3897/zookeys.778.24562

**Published:** 2018-08-02

**Authors:** Deneb Ortigosa, Nancy Yolimar Suárez-Mozo, Noe C. Barrera, Nuno Simões

**Affiliations:** 1 Unidad Multidisciplinaria de Docencia e Investigación Sisal (UMDI-SISAL), Facultad de Ciencias, Universidad Nacional Autónoma de México, Puerto de abrigo s/n, Sisal, CP 97356 Yucatán, Mexico Universidad Nacional Autónoma de MéxicoYucatánMexico; 2 Harte Research Institute, Texas A&M University-Corpus Christi, 300 Ocean Dr., Unit 5869, Corpus Christi, Texas 78412-5869, U.S.A. Texas A&M UniversityCorpus ChristiUnited States of America; 3 Laboratorio Nacional de Resiliencia Costera Laboratorios Nacionales, CONACYT, Mexico City, Mexico Laboratorio Nacional de Resiliencia Costera Laboratorios NacionalesMexico CityMexico; 4 International Chair for Coastal and Marine Studies, Harte Research Institute for Gulf of Mexico Studies, Texas A&M University – Corpus Christi, Corpus Christi, Texas, USA Texas A&M UniversityCorpus ChristiUnited States of America

**Keywords:** baseline, inventory, Campeche Bank, Gulf of Mexico, micromolluscs

## Abstract

Six sediment samples weighing between 224–735 g were collected in June of 2016 from Cayo Nuevo reef, located at the Campeche Bank, southern Gulf of Mexico. Samples were collected by SCUBA diving, from were two stations at depths of 7.6 and 18.2 m. Sediment was sieved and molluscs (adults and micromolluscs ≤ 10 mm) were sorted, examined, and identified to the lowest taxonomic level. A total of 1,347 specimens was found, of which 224 shells were dead and 1,123 were alive. Thirty-four families, 53 genera, and 67 species were identified. The most abundant families were Chamidae and Arcidae for the Bivalvia class, and Caecidae and Tornidae for the Gastropoda class. The vertical range of *Bentharca* sp. was extended.

## Introduction

Frequently, species molluscan biodiversity accounts are incomplete because of the lack of some groups such as sea slugs and micromolluscs. Compared with macromolluscs, the study of micromolluscs is still in its infancy, which is probably due not to the difficulty involved in obtaining samples, but difficulties in identification of such small animals and the time-consuming process required to separate specimens from sand or other substrates (e.g., algae or rocks), and photography. In order to get a more realistic picture of the biodiversity for different habitats, micromolluscs should be incorporated into the different studies (Sasaki 2008, and pers. obs.).

The term micromollusc has been applied in arbitrary and non-standardised ways. Micromolluscs are molluscs not visible without some type of artificial assistance, such as a microscope or magnifying glass. The most restrictive definition, or *sensu stricto*, stated that micromollusc size should be less than 5 mm as an adult (Narciso 2005, Geiger et al. 2007). Other authors considered micromolluscs as specimens smaller than 10 mm as an adult (Barrera and Tunnell 2001). Finally, the wider definition of micromollusc, or *sensu lato*, includes molluscs whose size is typically less than 10 mm as an adult and also included juvenile representative of macromolluscs (Moore 1964; García-Cubas 1970; Tunnell 1974; Kay 1980; Vokes and Vokes 1983).

Barrera (2001) stated that within Texas and Mexico, the majority of the studies involved macromolluscs. In Mexico, more than 4,643 species of marine molluscs have been recorded, and approximately 2,067 of them inhabit the Gulf of Mexico (GoMx) and the Mexican Caribbean Sea (Castillo-Rodríguez 2014). Unfortunately, Castillo-Rodríguez did not state which of them were micromolluscs. Important molluscan compilations focusing on these groups include publications by García-Cubas and Reguero (2007) and Vokes and Vokes (1983), although only a few publications have addressed the micromolluscan fauna specifically such as García-Cubas (1963, 1970 and 1971) for lagoons in the Gulf of Mexico and Hicks et al. (2001) at Alacranes reef.

**Figure 1.**
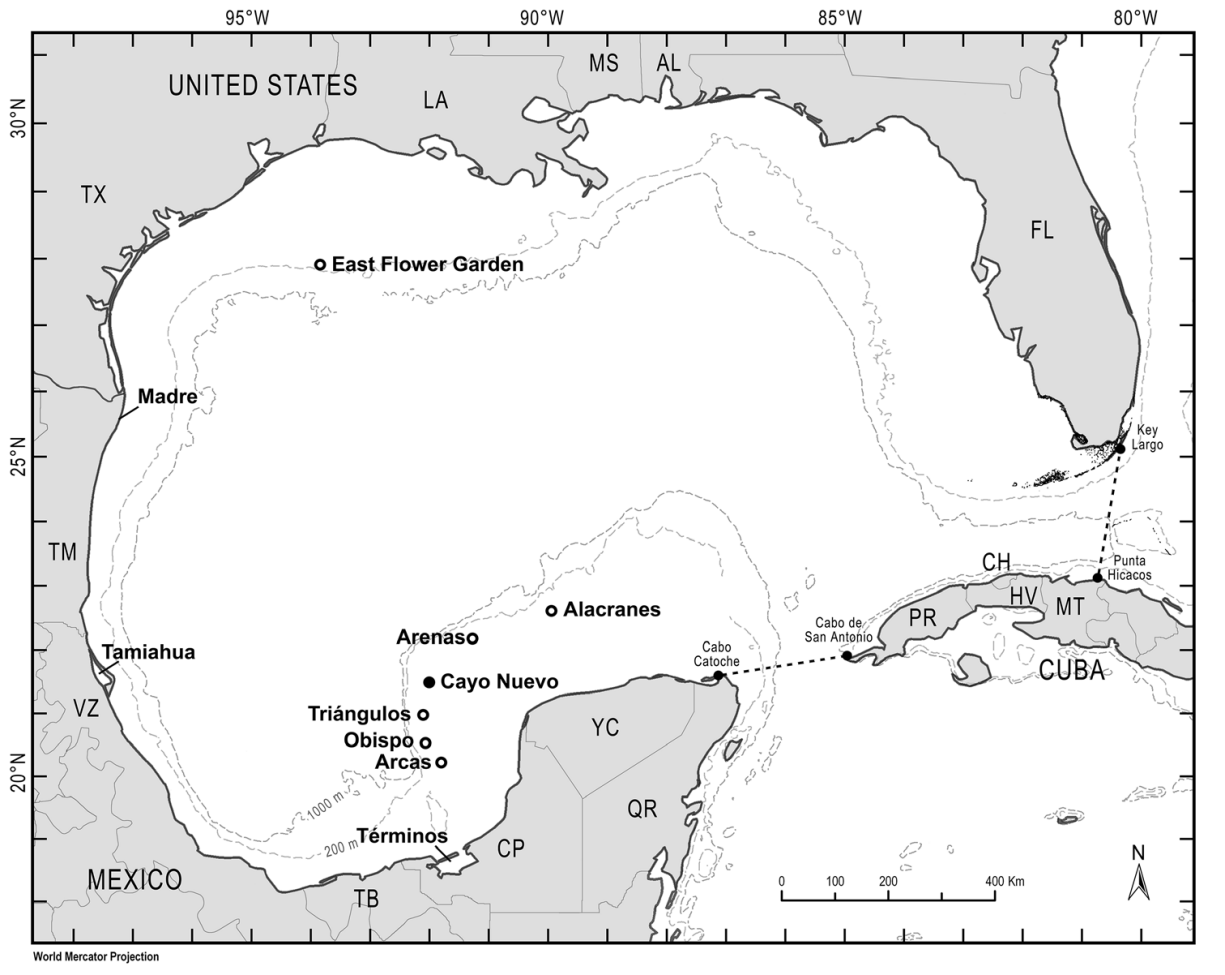
Map of the Gulf of Mexico, with the largest reef in the GoMx and sampling locations where micromolluscs have been documented in the literature and this study (adapted from Felder and Camp 2009).

The Campeche Bank is located at the southern GoMx and is composed of several emergent and submerged coral reefs (Tunnell et al. 2010). One of the smaller and most remote reefs is Cayo Nuevo, located between Arenas and Triángulos reefs (190 km offshore) in the GoMx (Fig. 1). Studies on this reef are almost non-existent with the exception of polychaetes (Granados-Barba et al. 2003).

The present work focuses on the molluscs of the Cayo Nuevo sandy bottoms, Gulf of Mexico. In this substrate we could find micromolluscs *sensu stricto* and juveniles of macromolluscs species that inhabit the interstitial as but also empty shells that could be carried by external factors such wind and currents.

## Materials and methods

Using SCUBA gear, six sediment samples of coarse sand to fine gravel weighing 224–735 g each were collected by hand at 7.6 and 18.2 m on 19 June 2017 at Cayo Nuevo reef (Table 1). Each sample was sieved by pouring water through six differently sized sieves (2 mm, 1.4 mm, 1 mm; 710 µm, 500 µm, and 250 µm) (Table 2) and sorted dry using a dissecting Nikon SMZ800 microscope. Specimens were picked out using soft forceps and 000 fine paint brushes. Molluscs were placed into 2 mm tubes and micromolluscs were placed into PCR tubes, both with 70% ethanol for long-term storage. Identification of specimens to species level was based on Abbott (1974), Tunnell et al. (2010) and Redfern (2013). Whenever possible, at least one specimen of each species or morphotype was photographed. All the specimens were deposited at the “Colección de Moluscos de la Peninsula de Yucatán” (**CMPY**), Unidad Multidisciplinaria de Docencia e Investigación Campus Sisal, Universidad Nacional Autónoma de México.

**Table 1.** Sampling stations and coordinates at Cayo Nuevo on 18 June 2016.

Station	Depth (m)	Latitude (N)	Longitude (W)	Sample
1	18.2	21°49'40.32", 92°4'37.62"		GoMex-001
				GoMex-002
				GoMex-002
				GoMex-004
2	7.6	21°49'47.82", 92°4'34.32"		GoMex-005
				GoMex-006

**Table 2.** Weight of each sample per sieve size in grams.

Sieve	GoMex-001	GoMex-002	GoMex-002	GoMex-004	GoMex-005	GoMex-006
2 mm	86.89	335.85	372.2	645.4	679.1	615.19
1.4 mm	46.94	60.04	21.7	46	80.7	91.53
1 mm	34.01	28.43	5.7	27.3	14.8	22.96
710 µm	27.1	11.13	1.3	28.5	2.3	5.32
500 µm	16.04	3.07	0.3	16.7	0.7	0.82
250 µm	13.86	1.2	0.07	2.6	0.6	0.004
Total	224.84	439.72	401.27	766.5	778.2	735.824

The nomenclature of the species listed was assigned according to Bouchet and Rocroi (2010) for Bivalvia, Bouchet et al. (2017) for Gastropoda, and Kaas and Van Belle (1985) for Polyplacophora, due to the variability of some categories we only present the Linnaean ones. Abundance categories were assigned following Hicks et al. (2001): Abundant ≥ 50 (A); Common = 6–49 (C); Uncommon = 2–5 (UC), and Rare = 1 (R). Juvenile species are denoted by an asterisk (*).

## Results

The results from the analysis of the sediment from Cayo Nuevo reef revealed 67 species of molluscs, from which 50 species are gastropods, 14 species are bivalves and three are chitons. These species belong to 38 different families.

**Phylum Mollusca Linnaeus, 1758**

**Class Polyplacophora Gray, 1821**

**Figure 2.**
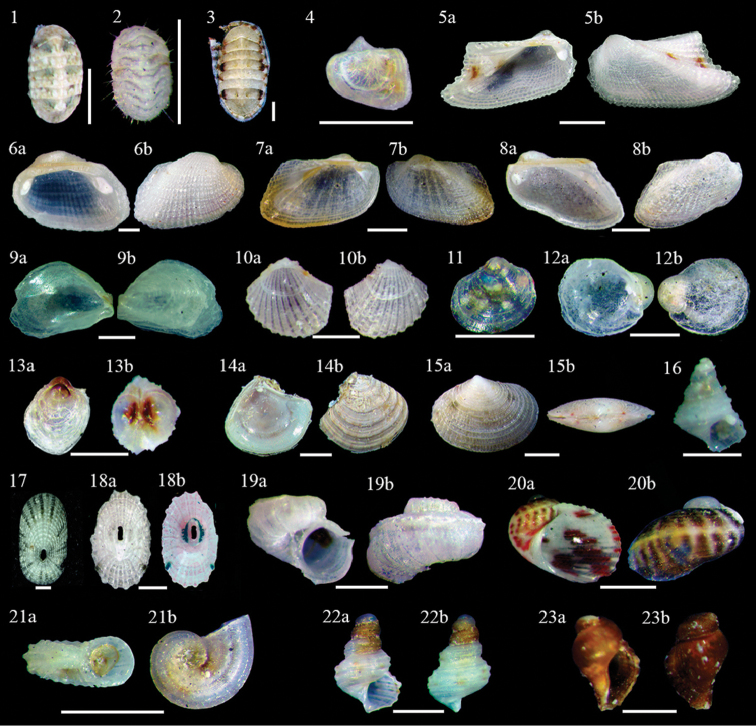
Polyplacophora**1–3**: **1**Polyplacophora sp. 1, dorsal view, scale bar 0.5 mm **2**Polyplacophora sp. 2, dorsal view, scale bar 1 mm **3***Ischnochiton* sp., dorsal view, scale bar 0.5 mm. Bivalvia**4–15**: **4**Arcidae sp., dorsal view, scale bar 0.5 mm **5***Arcaimbricata***5a** ventral view **5b** dorsal view, scale bar 1 mm **6***Barbatiadomingensis***6a** ventral view **6b** dorsal view, scale bar 1 mm **7***Barbatia* sp. **7a** ventral view **7b** dorsal view, scale bar 1 mm **8***Bentharca* sp. **8a** ventral view **8b** dorsal view, scale bar 1 mm **9***Anomia* sp. **9a** ventral view **9b** dorsal view, scale bar 1 mm **10***Carditopsissmithii***10a** ventral view **10b** dorsal view, scale bar 1 mm, **11**Lucinidae sp., ventral view, scale bar 0.5 mm **12***Chamasinuosa***12a** ventral view **12b** dorsal view, scale bar 0.5 mm **13**Chamidae sp. **13a** ventral view **13b** dorsal view, scale bar 1 mm, **14***Crassinellalunulata***14a** ventral view **14b** dorsal view, scale bar 1 mm **15***Semelebellastriata***15a** dorsal view **15b** lateral view, scale bar 1 mm. Gastropoda**16–23**: **16**Gastropoda sp., ventral view, scale bar 0.5 mm, **17***Diodoraminuta*, ventral view, scale bar 0.25, **18***Diodoralisteri***18a** ventral view **18b** dorsal view, scale bar 1 mm, **19***Scissurellaredferni***19a** ventral view **19b** dorsal view, scale bar 1 mm **20***Synaptocohleapicta***20a** ventral view **20b** dorsal view, scale bar 1 mm **21***Lodderenaornata***21a** ventral view **21b** Apical view, scale bar 0.5 mm **22***Cerithium* sp. 1 **22a** ventral view **22b** dorsal view, 1 scale bar 1 mm **23***Cerithium* sp. 2 **23a** ventral view **23b** dorsal view, scale bar 1 mm.

**Polyplacophora sp. 1** * (R) (Fig. 2–1)

**Polyplacophora sp. 2** * (R) (Fig. 2–2)

Order Chitonina Thiele, 1909

Family Chitonoidea Rafinesque, 1815

Genus *Ischnochiton* Gray, 1847

**Figure 3.**
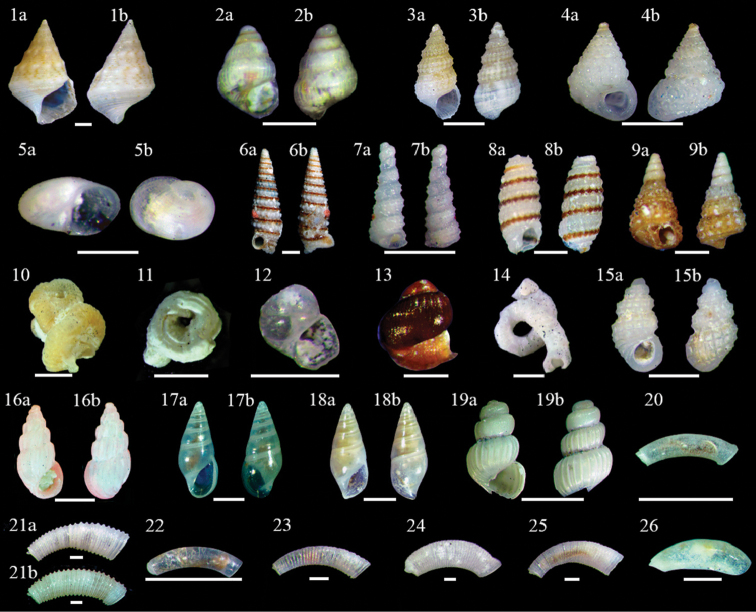
Gastropoda**1–26**: **1***Cerithiumatratum***1a** ventral view **1b** dorsal view, scale bar 1 mm **2***Alabaincerta***2a** ventral view **2b** dorsal view, scale bar 0.25 mm **3***Finella* sp. **3a** ventral view **3b** dorsal view, scale bar 1 mm **4***Sansoniatuberculata***4a** ventral view **4b** dorsal view, scale bar 1 mm **5***Hipponix* sp. **5a** ventral view **5b** dorsal view, scale bar 0.1 mm **6***Iniforisturristhomae***6a** ventral view **6b** dorsal view, scale bar 1 mm **7***Metaxiarugulosa***7a** ventral view **7b** dorsal view, scale bar 0.5 mm **8***Cerithiopsis* sp. **8a** ventral view **8b** dorsal view, scale bar 1 mm **9**Cerithiopsiscf.iuxtafuniculata**9a** ventral view **9b** dorsal view, scale bar 1 mm **10**Vermetidae*incertae sedis* irregularis scale bar 1 mm **11***Dendropomacorrodens* scale bar 1 mm **12** Vermetid sp. C, ventral view, scale bar 0.25 mm **13***Petaloconchusmcgintyi*, ventral view, scale bar 0.25 mm **14***Thylacodes* sp. scale bar 1 mm **15***Simulamerelinacaribaea***15a** ventral view **15b** dorsal view, scale bar 1 mm **16***Schwartziellafischeri***16a** ventral view **16b** dorsal view, scale bar 1 mm **17***Zebina* sp. 2 **17a** ventral view **17b** dorsal view, scale bar 1 mm **18***Zebina* sp. 2 **18a** ventral view **18b** dorsal view, scale bar 0.5 mm **19***Truncatella* sp. **19a** ventral view **19b** dorsal view, scale bar 0.5 mm **20***Caecumcircumvolutum*, lateral view, scale bar 0.2 mm **21***Caecumdonmoorei***21a** stage 1, lateral view, scale bar 0.2 mm **21b** adult, lateral view, scale bar 0.2 mm **22***Caecumjohnsoni*, lateral view, scale bar 0.2 mm **23***Caecumpulchellum*, lateral view, scale bar 0.2 mm **24***Caecumtextile*, lateral view, scale bar 0.2 mm **25***Caecum* sp. B, lateral view, scale bar 0.2 mm **26***Meiocerasnitidum*, lateral view, scale bar 0.2 mm.

***Ischnochiton* sp.** * (UC) (Fig. 2–3)

**Class Bivalvia Linnaeus, 1758**

Order Arcida Stoliczka, 1871

Family Arcidae Lamarck, 1809

**Figure 4.**
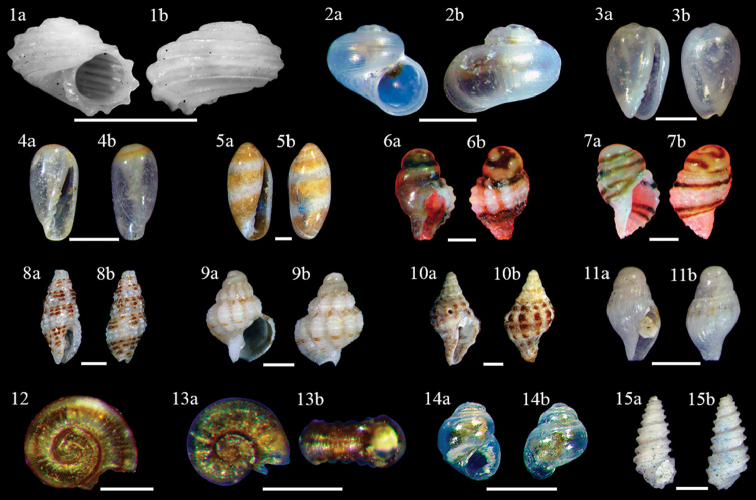
Gastropoda**1–18: 1***Parviturboides* sp. **1a** ventral view **1b** dorsal view, scale bar 1 mm **2***Vitrinella* sp. **2a** ventral view **2b** dorsal view, scale bar 0.5 mm **3***Gibberulalavalleeana***3a** ventral view **3b** dorsal view, scale bar 1 mm **4***Volvarina* sp. 1 **4a** ventral view **4b** dorsal view, scale bar 1 mm **5***Volvarina* sp. 2 **5a** ventral view **5b** dorsal view, scale bar 1 mm **6**Columbellidae sp. 1 **6a** ventral view **6b** dorsal view, scale bar 0.5 mm **7**Columbellidae sp. 2 **7a** ventral view **7b** dorsal view, scale bar 0.5 mm **8***Steironepionmoniferum***8a** ventral view **8b** dorsal view, scale bar 1 mm **9***Phrontis* sp. 9a ventral view **9b** dorsal view, scale bar 0.5 mm **10***Trachypollia* sp. **10a** ventral view **10b** dorsal view, scale bar 1 mm **11**Turridae sp. 1 **11a** ventral view **11b** dorsal view, scale bar 1 mm **12***Ammoniceralineofuscata*, Apical view, scale bar 0.25 mm **13***Ammoniceraminortalis***13a** Apical view **13b** ventral view, scale bar 0.1 mm **14***Rissoellagalba***14a** ventral view **14b** dorsal view, scale bar 0.1 mm **15**Pseudoscillaaff.babylonia**15a** ventral view **15b** dorsal view, scale bar 1 mm.

**Arcidae sp**. (UC) (Fig. 2–4)

Genus *Arca* Linnaeus, 1758

***Arcaimbricata* Bruguière, 1789** * (UC) (Fig. 2–5a, b)

Genus *Barbatia* Gray, 1842

***Barbatiadomingensis*** * (Lamarck, 1819) (UC) (Fig. 2–6a, b)

***Barbatia* sp.** * (C) (Fig. 2–7a, b)

Genus *Bentharca* Verrill & Bush, 1898

***Bentharca* sp.** (R) (Fig. 2–8a, b)

Order Mytilida Férussac, 1822

Family Mytilidae Rafinesque, 1815

Genus *Crenella* T. Brown, 1827

***Crenella* sp.** (R)

Order Pectinida Gray, 1854

Family Anomiidae Rafinesque, 1815

Genus *Anomia* Linnaeus, 1758

*Anomia* sp.* (R) (Fig. 2–9a, b)

Order Cardita Bruguière, 1792

Family Carditidae Férussac, 1822

Genus *Carditopsis* E. A. Smith, 1881

***Carditopsissmithii* (Dall, 1896)** (C) (Fig. 2–10a, b)

Order Lucinida Gray, 1854

Family Lucinidae J. Fleming, 1828

**Lucinidae sp.** * (UC) (Fig. 2–11)

Order Venerida Gray, 1854

Family Chamidae Lamarck, 1809

**Chamidae** sp. (UC) (Fig. 2–12a, b)

Genus *Chama* Linnaeus, 1758

***Chamasinuosa* Broderip, 1835** * (A) (Fig. 2–13a, b)

Family Galeommatidae Gray, 1840

**Galeommatidae sp.** (R)

Order Carditida Dall, 1889

Family Crassatellidae Férussac, 1822

Genus *Crassinella* Guppy, 1874

***Crassinellalunulata*** (Conrad, 1834) * (R) (Fig. 2–14a, b)

Order Cardiida Férussac, 1822

Family Semelidae Stoliczka, 1870 (1825)

Genus *Semele* Schumacher, 1817

***Semelebellastriata* (Conrad, 1837)** * (UC) (Fig. 2–15a, b)

**Class Gastropoda Cuvier, 1795**

**Gastropoda sp.** * (R) (Fig. 2–16)

Order Lepetelloidea Dall, 1882

Family Fissurellidae Fleming, 1822

Genus *Diodora* Gray, 1821

***Diodoraminuta* (Lamarck, 1822)** * (UC) (Fig. 2–17)

***Diodoralisteri* (d’Orbigny, 1847)** (R) (Fig. 2–18a, b)

Family Scissurellidae Gray, 1847

Genus *Scissurella* d’Orbigny, 1824

***Scissurellaredferni* (Rolán, 1996)** (C) (Fig. 2–19a, b)

Order Trochida Rafinesque, 1815

Family Trochidae Rafinesque, 1815

Genus *Synaptocochlea* Pilsbry, 1890

***Synaptocochleapicta* (d’Orbigny, 1847)** (A) (Fig. 2–20a, b)

Family Skeneidae W. Clark, 1851

Genus Lodderena Iredale, 1924

***Lodderenaornata* (Olsson & McGinty, 1958)** (A) (Fig. 2–21a, b)

Family Cerithiidae Fleming, 1822

Genus *Cerithium* Bruguière, 1789

***Cerithium* sp. 1** (R) (Fig. 2–22a, 22b)

***Cerithium* sp. 2** (UC) (Fig. 2–23a, 23b)

***Cerithiumatratum* (Borns, 1778)** (R) (Fig. 3–1a, b)

Family Litiopidae Gray, 1847

Genus *Alaba* H. adams & A. Adams, 1853

***Alabaincerta* (d’Orbigny, 1841)** (C) (Fig. 3–2a, b)

Family Scaliolidae Jousseaume, 1912

***Finella* sp.** (UC) (Fig. 3–3a, 3b)

Family Pickworthiidae Iredale, 1917

Subfamily Pickworthiinae Iredale, 1917

Genus *Sansonia* Jousseaume, 1892

***Sansoniatuberculata* (Watson, 1886)** (R) (Fig. 3–4a, b)

Family Hypponicidae Troschel, 1861

***Hipponix* sp.** (C) (Fig. 3–5a, 5b)

Family Triphoridae Gray, 1847

Genus *Iniforis* Jousseaume, 1884

***Iniforisturristhomae* (Holten, 1802)** (UC) (Fig. 3–6a, b)

Genus *Metaxia* Monterosato, 1884

***Metaxiarugulosa* (C. B. Adams, 1850)** (R) (Fig. 3–7a, b)

Family Cerithiopsidae H. Adams & A. Adams, 1853

Genus *Cerithiopsis* Forbes & Hanley, 1850

***Cerithiopsis* sp.** (R) (Fig. 3–8a, 8b)

**Cerithiopsiscf.iuxtafuniculata Rolán, Espinosa & Fernández-Garcés, 2007** (R) (Fig. 3–9a, b)

Family Vermetidae Rafinesque, 1815

**Vermetidae*incertae sedis irregularis* d’Orbigny, 1841** (Fig. 3–10)

Genus *Dendropoma* Mörch, 1861

***Dendropomacorrodens* (d’Orbigny, 1841)** (R) (Fig. 3–11)

**Vermetid sp. C**Redfern 2013 (A) (Fig. 3–12)

Genus *Petaloconchus* Lea, 1843

***Petaloconchusmcgintyi* (Olsson & Harbison, 1953)** * (C) (Fig. 3–13)

Genus *Thylacodes* Guettard, 1770

***Thylacodes* sp.** * (R) (Fig. 3–14)

Family Rissoidae Gray, 1847

Genus *Simulamerelina* Ponder, 1985

***Simulamerelinacaribaea* (d’Orbigny, 1842)** (UC) (Fig. 3–15a, b)

Family Zebinidae Coan, 1964

Genus *Schwartziella* G. Nevill, 1881

***Schwartziellafischeri* (Desjardin, 1949)** (UC) (Fig. 3–16a, b)

Genus *Zebina* H. Adams & A. Adams, 1854

***Zebina* sp. 1** (A) (Fig. 3–17a, 17b)

***Zebina* sp. 2** (C) (Fig. 3–18a, 18b)

Family Truncatellidae Gray, 1840

Genus Truncatella Risso, 1826

***Truncatella* sp.** (R) (Fig. 4–19a, 19b)

Family Caecidae Gray, 1850

Genus *Caecum* Fleming, 1813

***Caecumcircumvolutum* de Folin, 1867** (C) (Fig. 3–20)

***Caecumdonmoorei* Mitchell-Tapping, 1979** (C) (Fig. 3–21a, b)

***Caecumjohnsoni* Winkley, 1908** (A) (Fig. 3–22)

***Caecumpulchellum* Stimpson, 1851** (C) (Fig. 3–23)

***Caecumtextile* de Folin, 1867** (UC) (Fig. 3–24)

***Caecum* sp. B *sensu*Redfern 2013** (C) (Fig. 3–25)

Genus *Meioceras* Carpenter, 1859

***Meiocerasnitidum* (Stimpson, 1851)** (UC) (Fig. 3–26)

Family Tornidae Sacco, 1896 (1884)

Genus *Parviturboides* Pilsbry & McGinty, 1949

***Parviturboides* sp.** (C) (Fig. 4–1a, b)

Genus *Vitrinella* C. B. Adams, 1850

***Vitrinella* sp.** (A) (Fig. 4–2a, b)

Family Cystiscidae Stimpson, 1865

Genus *Gibberula* Swainson, 1840

***Gibberulalavalleeana* (d’Orbigny, 1824)** (UC) (Fig. 4–3a, b)

Family Marginellidae Fleming, 1828

Genus *Volvarina* Hinds, 1844

***Volvarina* sp. 1** (UC) (Fig. 4–4a, 4b)

***Volvarina* sp. 2** (R) (Fig. 4–5a, 5b)

Family Columbellidae Swainson, 1840

**Columbellidae sp. 1** (UC) (Fig. 4–6a, b)

**Columbellidae sp. 2** (R) (Fig. 4–7a, 7b)

Genus *Steironepion* Pilsbry & H. N. Lowe, 1932

***Steironepionmoniliferum* (G. B. Sowerby I, 1844)** (UC) (Fig. 4–8a, b)

Family Nassariidae Iredale, 1916 (1835)

Genus *Phrontis* H. Adams & A. Adams, 1853

***Phrontis* sp.** (UC) (Fig. 4–9a, b)

Family Muricidae Rafinesque, 1815

Genus *Trachypollia* Woodring, 1928

***Trachypollia* sp.** (R) (Fig. 4–10a, b)

Family “Turridae” H. Adams & A. Adams, 1853 (1838)

**Turridae sp. 1** (R) (Fig. 4–11a, 11b)

Family Omalogyridae G.O. Sars, 1878

Genus *Ammonicera* Vayssière, 1893

***Ammoniceralineofuscata* Rolán, 1992** (A) (Fig. 4–12)

***Ammoniceraminortalis* Rolán, 1992** (A) (Fig. 4–13a, b)

Family Rissoellidae Gray, 1850

Genus Rissoella Gray, 1847

***Rissoellagalba* Robertson, 1961** (R) (Fig. 4–14a, b)

Order Aplysiida

Family Aplysiidae Lamarck, 1809

Genus *Aplysia* Linnaeus, 1767

***Aplysia* sp.** (R)

Order Siphonarimorpha

Family Pyramidellidae Gray, 1840

Genus *Pseudoscilla* Boettger, 1901

**Pseudoscillaaff.babylonia (C. B. Adams, 1845)** (R) (Fig. 4–15a, 185b)

## Discussion

The most abundant families of gastropods were the Caecidae (456 specimens, seven species), Tornidae (221 specimens, two species), and Omalogyridae (132 specimens, two species). The most abundant families of bivalves were Arcidae (40 specimens, five species) and Chamidae (59 specimens, two species). The most abundant gastropod species were *Caecumjohnsoni* (310 specimens), *Vitrinella* sp. (208 specimens), Vermetid sp. C (91 specimens), *Lodderenaornata* (71 specimens), and *Caecumdonmoorei* (147 specimens). For the Bivalvia the most abundant species were *Chamasinuosa* (57 specimens), *Barbatiadomingensis* (57 specimens) and *Carditopsissmithii* (12 specimens).

From the six sediment samples, the most commonly found molluscs were *Lodderenaornata*, *Caecumjohnsoni* and *Ammoniceralineofuscata*, while other species appeared only once: *Leptochiton* sp., Arcidae sp., *Bentharca* sp., *Crenella* sp., *Anomia* sp., *Chama* sp., Galeommatidae sp., *Chioneelevata*, *Semelebellastriata*, *Cerithium* sp. 1, Gastropoda sp., *Diodoralisteri*, *Cerithiumatratum*, *Sansoniatuberculata*, *Iniforisturristhomae*, *Metaxiarugulosa*, Cerithiopsiscf.iuxtafuniculata, *Cerithiopsis* sp., Vermetidae*incertae sedis* irregularis, *Dendropomacorrondens*, *Thylacodes* sp., *Finella* sp., *Caecumtextile*, *Hipponix* sp., *Volvarina* sp. 2, Columbellidae sp. 2, *Aplysia* sp., and Pseudoscillaaff.babylonia.

This new data becomes a taxonomic reference list for the molluscs that inhabit Cayo Nuevo, GoMx, including micromolluscs as well as juvenile macromolluscs. To place it within a useful context we mention other inventories made in this area: Felder and Camp (2009) recorded some 5,517 species of invertebrates in the GoMx, of which 2,455 were marine molluscs (Moretzsohn et al. 2009). González et al. (1991) recorded 298 species of molluscs and included 33 localities distributed around the coasts of the Yucatan Peninsula and adjacent coral reefs but did not mentioned Cayo Nuevo. García-Cubas et al. (1999) recorded 110 species of gastropods in the northern and northeastern regions of the Yucatan Peninsula. Rice and Kornicker (1962) recorded 130 species for Alacranes reef in the Campeche Bank and later, Hicks et al. (2001) recorded 215 species of molluscs on the same reef. Although earlier articles include reefs or sampling locations within the Bank of Campeche (e.g., Rehder and Abbott 1951, Springer and Bullis 1956, Kornicker et al. 1959), no mention of molluscs from Cayo Nuevo were found. Only Barrera (2001) study focuses on reef micromolluscs, recording 131 species from the East and West Flower Garden Banks (FGB).

The molluscan assemblage at Cayo Nuevo shares many species also present at the FGB (Barrera 2001) and Alacranes Reef (Hicks et al. 2001) (19 families/21 genera and 21 families/22 genera, respectively) (Table 3). The most diverse families recorded by Barrera (2001) and Hicks et al. (2001) were Caecidae (six genera and ten species) and Rissoidae (five genera and seven species) for Gastropoda and Arcidae (four genera and seven species) for Bivalvia.

**Table 3.** Comparison of molluscs recorded at Cayo Nuevo (present study) and other interstial records at the GoMx; Madre: García-Cubas (1970), Tamiahua: García-Cubas (1971), Términos: García-Cubas (1963, 1981), East Flower Garden: Barrera and Tunnell (2001), Alacranes: Hicks et al. (2001). The maximum recorded size is provided: Key: a) García-Cubas and Reguero (2004), b) Tunnell et al. (2010), c) Redfern (2013).

Systematics	Lagoons			Reefs			Maximum recorded size (mm)
	Madre	Tamiahua	Términos	East Flower Garden	Alacranes	Cayo Nuevo	
**Class Bivalvia**							
* Arca imbricata *			*		*	*	48^c^
* Barbatia domingensis *				*	*	*	30^b^
* Carditopsis smithii *				*	*	*	1.5^c^
* Chama sinuosa *					*	*	76^b^
* Crassinella lunulata *		*				*	8^b^
* Semele bellastriata *						*	14^c^
**Class Gastropoda**							
* Diodora minuta *						*	10.5^c^
* Diodora listeri *					*	*	45^b^
* Scissurella redferni *						*	1^c^
* Synaptocochlea picta *					*	*	3.5^c^
* Lodderena ornata *				*	*	*	0.8^c^
* Cerithium atratum *						*	
* Alaba incerta *						*	
* Sansonia tuberculata *						*	1.5^c^
* Iniforis turristhomae *				*	*	*	6^c^
* Metaxia rugulosa *						*	7^c^
Cerithiopsis cf. iuxtafuniculata						*	3^c^
Vermetidae *incertae sedis irregularis*						*	6^b^
* Dendropoma corrodens *					*	*	10^b^
* Petaloconchus mcgintyi *				*	*	*	35^b^
* Simulamerelina caribaea *						*	3^b^
* Schwartziella fischeri *						*	3.5^c^
* Caecum circumvolutum *						*	4^b^
* Caecum donmoorei *						*	2^c^
* Caecum johnsoni *			*	*		*	5^b^
* Caecum pulchellum *	*	*	*	*	*	*	2^a^
* Caecum textile *					*	*	2^b^
* Meioceras nitidum *		*	*			*	3^b^
* Gibberula lavalleeana *		*				*	4^b^
* Steironepion moniliferum *						*	
* Ammonicera lineofuscata *						*	0.6^c^
* Ammonicera minortalis *				*		*	0.5^c^
* Rissoella galba *				*		*	1^c^
Pseudoscilla aff. babylonia				*	*	*	3^b^

Barrera (2001) most abundant gastropod species were *Amphithalamusvallei* (672 individuals) and cf. *Vitrinella* sp. (534 individuals); however, at Cayo Nuevo, 208 individuals of sf. *Vitrinella* sp., were found. Differences in the numbers of collected individuals can be explained by geography, but also by differences in the quantity of sediment collected and processed, fifteen sites with 300 ml sediment sampled at FGB and six samples of 224–735 g at Cayo Nuevo. *Vitrinella* sp. could not be identified to specific level due to the low similarity of characters shown with other described western Atlantic species. Barrera (2001) previously suggested that it could be an undescribed new species, but further detailed studies are required to establish its identity.

Regarding bivalves, the most abundant species reported by Barrera (2001) for the FGB were *Gregariellacoralliophaga* (145 individuals) (summing nine identified as *Barbatiadomingensis* (102 individuals) and eleven identified as *Barbatiacancellaria* (68 individuals, currently a synonym), and *Carditopsissmithii* (51 individuals). In comparison, 37 individuals of *Barbatiadomingensis* and 12 *Carditopsissmithii* were collected at Cayo Nuevo and these were not the most abundant species.

It should come as no surprise that many organisms were not identified to species level (e.g., *Leptochiton* sp., *Bentharca* sp., *Crenella* sp., *Anomia* sp., *Lottia* sp., *Diodora* sp., *Cerithium* sp., *Cerithiopsis* sp., *Thylacodes* sp., *Zebina* sp. 1, *Zebina* sp. 2, and *Phrontis* sp.). We relied on regional and local literature that in fact was scarce. In the case of juveniles, shells within a genus are similar because they share many characters and the differential characters are difficult to discern even as adults and almost impossible in juveniles. Our specimens identified under the name of *Gibberulalavalleeana* could be considerate as a species complex, due to the evidence and description of new species in Cuban waters (Espinosa and Ortea 2007).

These faunistic results from Cayo Nuevo represent the first inventory of molluscs from this remote reef. These findings contribute to record expansions for the southern GoMx of *Bentharca* sp. This contribution highlights the importance of conserving small areas that can harbour a considerable diversity of organisms. Seasonal changes on the mollusc community assemblages were not evaluated but would be an interesting future project, as would collecting growth series of species to assist in confirming identifications. In 2004, González and Torruco stated the importance of the Campeche Bank’s reefs and proposed a marine reserve for the reefs located within this area, Cayo Nuevo included. However, this proposal never materialized and, up to now, only Alacranes reef has governmental protection under the status of marine reserve. Species checklists of micromolluscs, as well as other faunal groups, are of vital importance to serve as a baseline data set, due to the proximity to Mexico’s offshore oil production area within the GoMx. The soft benthic interstitial mollusc communities are diverse, and their monitoring could well represent ecological indicators of ecosystem health, especially in the light of potential future oil-spills.

## Supplementary materials

Supplementary material 1 Species abundance per sample Data type: occurences This is an open access article distributed under the terms of the Creative Commons Attribution License (CC BY 4.0), which permits unrestricted use, distribution, and reproduction in any medium, provided the original author and source are credited.Deneb Ortigosa, Nancy Yolimar Suárez-Mozo, Noe C. Barrera, Nuno Simões
